# A Unique Case of Unilateral Oculomotor Nerve Palsy Secondary to Dengue Fever

**DOI:** 10.7759/cureus.35281

**Published:** 2023-02-21

**Authors:** Sue Anne Loh, Wan Mohd Hafidz Wan Abdul Rahman, Khairy Shamel Sonny Teo, Norlelawati Abu

**Affiliations:** 1 Department of Ophthalmology and Visual Science, School of Medical Sciences, Health Campus, Universiti Sains Malaysia, Kubang Kerian, MYS; 2 Department of Ophthalmology, Hospital Tuanku Ja’afar, Seremban, MYS

**Keywords:** dengue-associated cranial neuropathy, cranial mononeuropathy, third nerve palsy, oculomotor nerve palsy, dengue fever

## Abstract

A wide range of ocular complications may arise from the mosquito-borne illness, dengue fever. We report a case of isolated unilateral oculomotor nerve palsy due to complications of dengue fever. A 50-year-old male with serologically confirmed dengue fever presented with a sudden onset of double vision with left eyelid drooping and left eye outward deviation on his day 8 of illness. Ocular examination revealed binocular diplopia with complete left eye ptosis and restriction of all left eye movements except for abduction. His left eye pupil was 8 mm dilated with a negative relative afferent pupillary defect (RAPD). A clinical diagnosis of left eye oculomotor nerve palsy with pupil involvement was established. Urgent contrasted brain imaging tests were performed and revealed to be normal. He was managed conservatively and had complete resolution of symptoms with good vision recovery within 3.5 months. Cranial mononeuropathy may be one of the various complications following dengue fever, as demonstrated in this case report. As it is an uncommon presentation, there is a need to exclude other acute causes of cranial nerve palsy. Visual prognosis is still favorable with judicious monitoring and without any treatment of steroids or immunoglobulin.

## Introduction

Dengue fever is endemic worldwide, affecting more than 100 countries, especially in Southeast Asia, including Bangladesh, Malaysia, the Philippines, Vietnam, and Indonesia [1]. Dengue fever in Malaysia recorded 397.71 cases per 100,000 populations in 2020, still accounting for the highest incidence rate among communicable diseases [2]. This mosquito-borne viral infection is a multisystemic disease that includes the ocular system. Dengue-related ocular manifestations are more confined to the posterior segment, and neuro-ophthalmic syndromes are rare, even more so for cranial nerve mononeuropathy [3]. We report an unusual sequela of isolated unilateral oculomotor nerve palsy following dengue fever.

This article was previously presented as a poster at the 14th Asia-Pacific Vitreo-Retina Society (APVRS) Congress on December 11-12, 2021.

## Case presentation

A 50-year-old male with underlying hypertension presented with a five-day history of high-grade fever, lethargy, and nausea. Both his dengue nonstructural protein 1 antigen and dengue-specific immunoglobulin results were positive. He was clinically diagnosed with dengue fever in the critical phase with warning signs.

**Figure 1:**
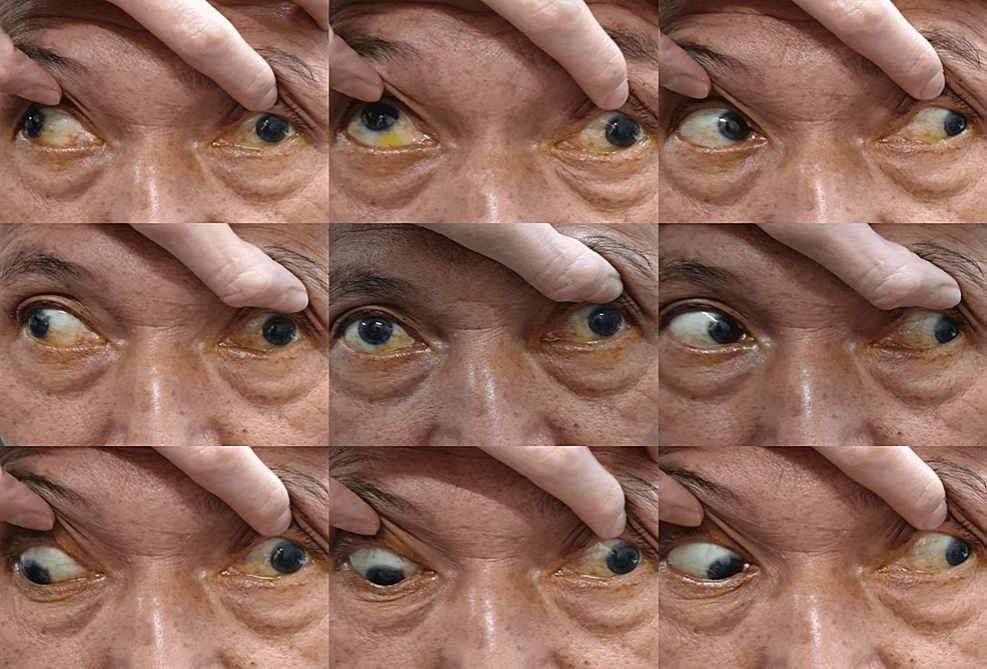
Limitation of all extraocular movement of the left eye except abduction during the initial presentation.

**Figure 2:**
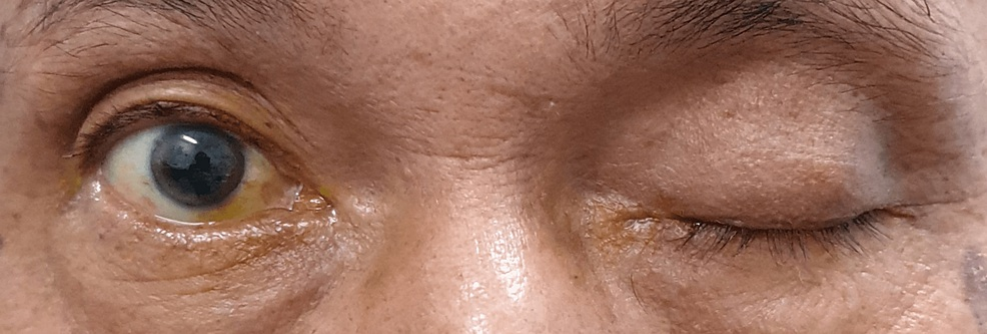
Initial presentation with complete ptosis of the left eye.

**Figure 3:**
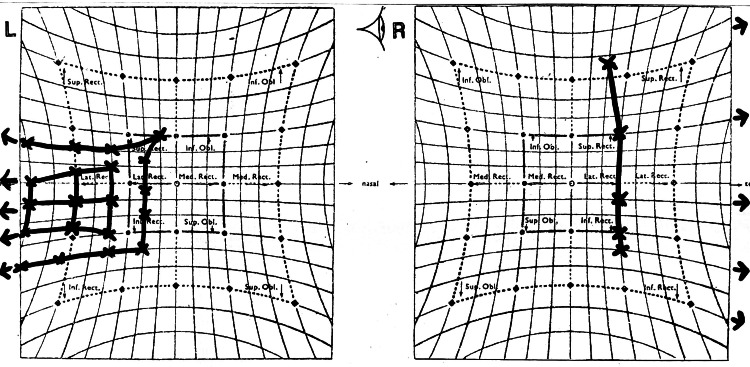
The Hess chart corresponds to the limitation of all extraocular movement of the left eye except abduction.

On day 8 of the illness, he had a sudden onset of double vision with left eyelid drooping and left eye outward deviation. On examination, his right eye visual acuity was 6/9, and his left eye visual acuity was 6/36, not improving with pinhole. He also complained of diplopia at all gazes. Complete left eye ptosis and restriction of all left eye movements except abduction were noted (Figure 1 and Figure 2). The Hess chart of his left eye showed restriction in all gaze except abduction, suggestive of oculomotor nerve palsy (Figure 3). His left eye pupil was 8 mm dilated, while his right eye pupil was 3 mm, and relative afferent pupillary defect (RAPD) was negative. Otherwise, both eye anterior segment, intraocular pressure, and posterior segment were normal. All these point toward a clinical diagnosis of left eye oculomotor nerve palsy.

His laboratory investigations during symptom presentation showed hemoconcentration (45%-52%) and thrombocytopenia (60-100 × 10^9^/L). He also developed transaminitis with elevated liver enzymes.

Urgent contrast-enhanced computed tomography (CECT) and computed tomography arteriography and venography (CTA/CTV) of the brain was done. Imaging results were revealed to be normal.

He was treated supportively with intravenous fluids and antipyretic. His hemodynamic state was stable throughout his hospital stay. We managed his oculomotor nerve palsy conservatively without any medical treatment.

**Figure 4:**
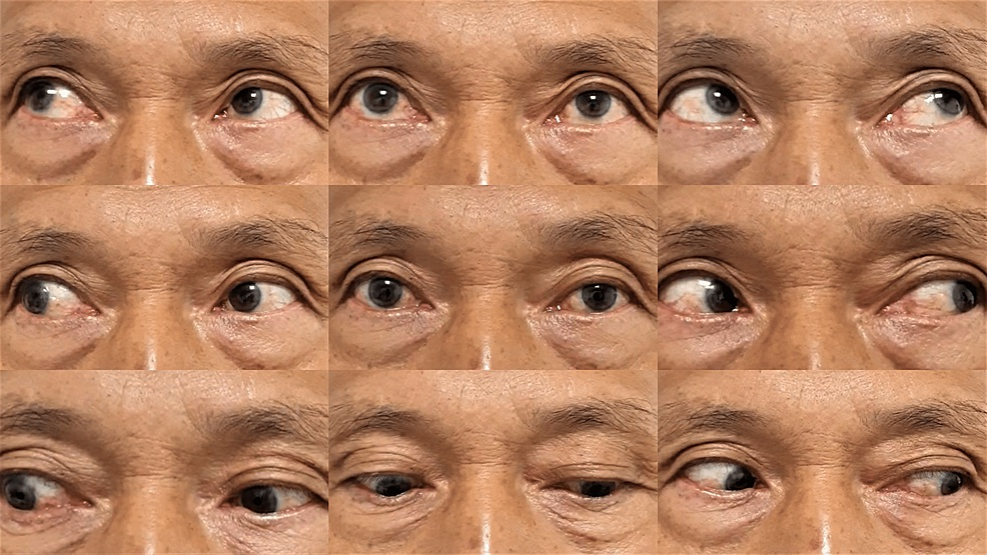
Improved ptosis and left eye gazes.

**Figure 5:**
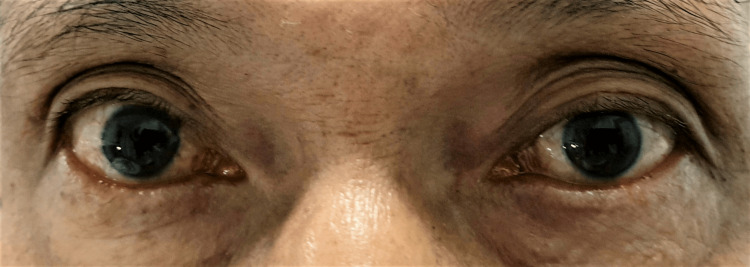
Resolution of the left eye ptosis.

**Figure 6:**
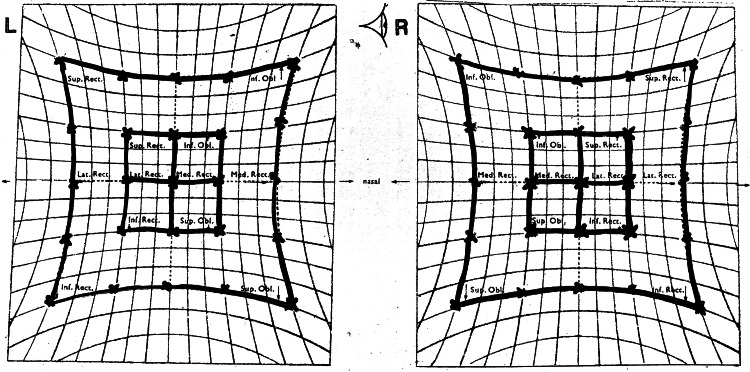
Improvement of left eye extraocular muscle movements.

His ocular symptoms persist during discharge, and he was asked to seek medical attention if any worsening of symptoms occurs. We monitor this patient frequently and during the first and second months of clinical visits, but his ocular symptoms persist. Not until the third month of the clinical visit, his ocular symptoms improved with vision recovery to 6/9 and resolution of eye movement limitation (Figures 4-6).

## Discussion

Dengue infection is an endemic arboviral disease that still imposes a global public health concern, especially in Southeast Asian countries. It is a vector-borne disease transmitted by *Aedes aegypti* mosquitoes with any of the four dengue viruses (DENV) serotypes: DENV-1, DENV-2, DENV-3, and DENV-4. DENV-2 and DENV-3 serotypes are commonly associated with neurological manifestations [4]. The prevalence of neurological manifestations of dengue fever is between 0.5% and 5.4% among Southeast Asians, whereby dengue-associated cranial neuropathy, including oculomotor nerve involvement, is considered rare [4]. Other ocular manifestations of dengue infection may include maculopathy, retinal edema, retinal hemorrhages, optic neuropathy, and vitritis [5].

The exact pathogenesis of dengue-associated cranial neuropathy is unknown, but it is thought to be immune-mediated. Dengue virus is a neurotropic virus that may cross the blood-brain barrier and replicate in the central nervous system. It would then directly infect endothelial cells, dendritic cells, and monocytes, which trigger cytokine overproduction, resulting in immune-mediated endothelial dysfunction [6]. The delayed onset of visual symptoms up to one week following dengue infection favors this immune-mediated theory, which is consistent with our patient, who had symptoms on day 8 after the onset of dengue fever [4].

Having said that, it is also important to eliminate other plausible causes of oculomotor motor palsy. CECT, CTA, and CTV brain examinations on our patient ruled out ischemic or hemorrhagic tumors and lesions, aneurysms, and vascular and cavernous sinus thrombosis.

The management of dengue-related cranial neuropathy is mainly conservative and supportive, which has been previously reported to improve without steroids or intravenous immunoglobulin [3,7-9]. Likewise, our patient recovered within three months postinfection without any medications and was advised for an eye patch for diplopia relief. The usage of steroid and immunoglobulin as treatment were reported, but mainly in cases of dengue-related maculopathy, optic neuropathy, and vasculitis [10]. The commencement of steroid therapy must be considered cautiously, as its usage may cause a rise in viral replication, especially during the febrile phase, which may lead to the worsening of the illness.

The overall prognosis for dengue-related ocular complications is good, and complete recovery coincides with improved platelet counts akin to our patient.

## Conclusions

Cranial mononeuropathy may be one of the various clinical features of dengue fever, as demonstrated in this case report. However, other causes of cranial nerve palsy need to be excluded as it is an uncommon presentation. Visual prognosis is still favorable with judicious monitoring and without any treatment of steroids or immunoglobulin.
